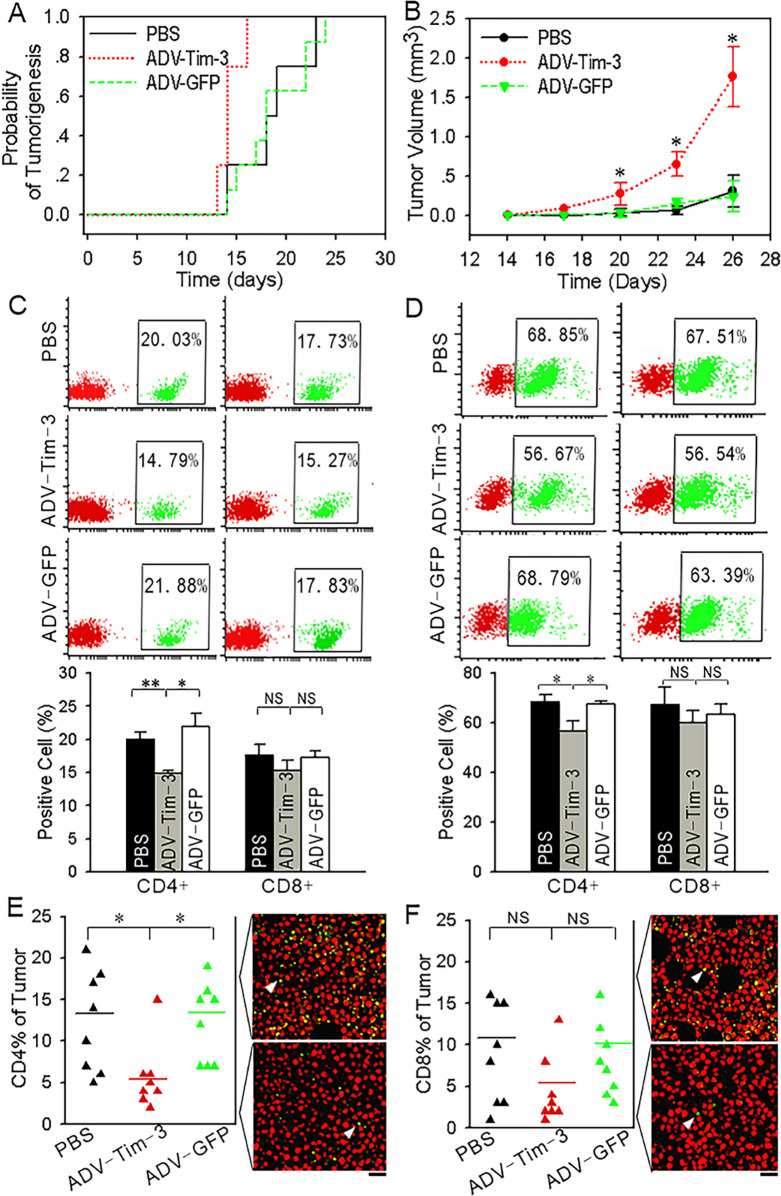# Correction: Lymphoma endothelium preferentially expresses Tim-3 and facilitates the progression of lymphoma by mediating immune evasion

**DOI:** 10.1084/jem.2009039703042021c

**Published:** 2021-03-15

**Authors:** Xiaoyuan Huang, Xiangyang Bai, Yang Cao, Jingyi Wu, Mei Huang, Duozhuang Tang, Si Tao, Tao Zhu, Yanling Liu, Yang Yang, Xiaoxi Zhou, Yanxia Zhao, Mingfu Wu, Juncheng Wei, Daowen Wang, Gang Xu, Shixuan Wang, Ding Ma, Jianfeng Zhou

Vol. 207, No. 3 | 10.1084/jem.20090397 | February 22, 2010

The authors regret that the ADV-GFP CD4^+^ image in Fig. 7 D was incorrect due to an error made during manuscript preparation. This error does not change the legend or affect the scientific conclusion of the published work. The corrected figure is shown here.

**Figure 7:**